# Health technology assessments conducted in health care facilities: A strategic practice? Findings from a content analysis of HTA reports

**DOI:** 10.1371/journal.pone.0185183

**Published:** 2017-09-25

**Authors:** Mathieu Ouimet, Pascal Lalancette, Alexandre Racine

**Affiliations:** Department of Political Science, Faculty of the Social Sciences, Université Laval, Québec, Québec, Canada; University of Tennessee Health Science Center, UNITED STATES

## Abstract

In this paper, we test the hypothesis that health technology assessment units located in hospitals tend to be more optimistic toward technologies that are currently in use in their organization than technologies that are not. The data include 108 health technologies assessed in 87 full-scale health technology assessment reports produced by the four main local health technology assessment units in Quebec (Canada) on behalf of decision makers from the same facility. We found that 58 (53.7 percent) of the 108 technologies were currently in use within the hospital during their assessment. Based on the assessors' interpretation of the scientific evidence regarding the efficacy of the technologies, 67.3 percent of the technologies that were in use in the hospital during the evaluation were effective (56 percent for those that were not currently being used), but the difference is not statistically significant (chi-square 1.38; p = 0.24). Controlling for the efficacy judgment, the type of technologies (i.e. preventive, diagnostic, therapeutic or organizational), the number of technologies assessed in the report and the assessment unit, we found that the technologies that were currently in use in the facility during the evaluation were 62 percent more likely to be recommended favorably by the assessment unit than the technologies that were not currently being used (RR = 1.62; 95 percent CI = 1.06–1.88). This suggests that the local health technology units that were examined in the study tended to be more optimistic toward technologies that were currently in use in their hospital at the time of the evaluation.

## Introduction

Local health technology assessment units (LHTAU) that are located in health care facilities bear the responsibility for performing health technology assessments to support evidence-informed decision making within their organization. LHTAUs systematically review the scientific evidence about the efficacy of various types of technologies and produce recommendations regarding whether or not the facility should be using the technology. Being in the business of scientific advice, theoretically, LHTAUs are considered independent and supposedly do not work under political influences. Otherwise, their scientific reputation would lower and their reports seen as being politicized. However, insights from the principal-agent (or agency) theory[1] suggest that LHTAUs might not be protected against influences. These insights are empirically proven in the case of provincial (non-local) health technology assessments agencies in Quebec: between January 1 and April 25, 2017, according to the Quebec lobbyist registry that is available online[2], formal mandates were obtained by 60 and 47 lobbyists to lobby the *Institut national d'excellence en santé et en services sociaux* and the *Institut national de santé publique du Québec*, respectively. Unfortunately, the registry does not collect lobbying data at the level of local health technology assessment units.

While to the best of our knowledge the principal-agent theory has not yet been used to prognosticate the direction of the recommendations made by LHTAUs, this theory was mobilized to examine scientific recommendations regarding the listing or delisting of carcinogens[3]. Principal-agent theory is useful for examining how a strategic principal delegate functions to a scientific adviser, and the strategies that the scientific adviser adopts to show he/she is fulfilling the functions competently.[3] Principal-agent theory applies in situations where it is too costly for the principal to verify closely how the agent behaves and when the principal and the agent have different utilities (i.e. satisfaction that gives to the actor a gain *x* if a state of the world is realized).

We know from game-theoretic experimental studies that an agent can sometimes use strategies such as language inflation or over-communication as a reaction to her estimation of the degree of misalignment between her preferences and those of the decision maker.[4–6] The main idea behind this paper is that a local health technology assessment unit is a strategic agent that might sometimes rely on similar strategies when producing its reports. We can also consider that the decision maker (the principal) also acts strategically when she chooses the in-house LHTAU to do a technology assessment. It is assumed in this study that in doing so, a decision maker is not only soliciting knowledge, but also legitimizing or delegitimizing arguments.

This study aims to test the hypothesis that the use of health technologies is more likely to be recommended positively by LHTAUs when the technologies are already in use inside the health care facility, irrespective of the judgment made by LHTAUs regarding their efficacy. This hypothesis has been deductively formulated based on the principal-agent theory[1] and was formulated before data collection. Following the standard model of scientific explanation [7,8], the research hypothesis can be seen as a prognosis that is based on (1) initial conditions and (2) theoretical and empirical regularities. Taken together, (1) and (2) form the *explanans*, while the prognosis (hypothesis) is the *explanadum*.

### Initial conditions

First, the LHTAU (the agent) and the decision maker who is asking for a technology assessment (the principal) are located in the same organization (i.e. a health care facility). Therefore, the LHTAU is not structurally or institutionally independent of the decision maker. Second, some of the technologies that the LHTAU is asked to evaluate are already in use in the facility, while others are not. This initial condition is crucial to our theoretical prediction, because it is the condition that directly makes our research hypothesis testable. Third, the LHTAU produces a report following a *voluntary* demand formulated by a decision maker who wishes to receive a recommendation regarding the relevance of using a health technology. As a consequence, the existence and survival of the LHTAU directly depend on its ability to stimulate the demand for its evaluation services within the facility. To stimulate the demand for its evaluation services, the evaluation unit needs to produce HTA reports that include recommendations sufficiently aligned with the preferences of the decision maker. Otherwise, the perceived costs of demanding a technology assessment would be higher, and the demand for the service would ultimately decline. In other words, the LHTAU is structurally incentivized to formulate recommendations that meet the decision maker's utility. The LHTAUs considered in this study can make decision makers reveal their preferences through conversations during the production phase or by asking them to comment on preliminary versions of their reports.

We can consider decision makers as having a multi-attribute utility function, meaning that a given HTA can procure their satisfaction for two main reasons, that is, by increasing their knowledge about a technology and by providing them with arguments to legitimize or delegitimize its use. Like the decision maker (the principal), the LHTAU (the agent) can also be seen as having a multi-attribute utility function. We posit that this utility function is composed of two main attributes, each corresponding to a reputational asset that is needed to survive as a local HTA unit. First, an HTA mandate provides the agent with the opportunity to increase its scientific reputation. In effect, to stimulate the demand for its evaluation services, the evaluation unit needs to produce HTA reports that are seen as coherent and rigorous. For example, in an HTA report that recommends favorably the use of a given technology, the authors of the report must have concluded that the technology is effective based on a systematic review of existing research evidence. Second, an HTA mandate provides the agent with the opportunity to increase its reputation of relevance or usefulness, a reputational asset that should be distinguished from the scientific reputation. A scientifically sound HTA that concludes that a technology currently in use in a facility is significantly outperformed by an alternative one could be perceived by a decision maker as a constraint given that the replacement of the technology is likely to be unacceptable in a given unit for various reasons such as when the technology is an important share of the practitioners’ revenues. Therefore, through their assessment reports, LHTAUs play their scientific and relevance reputation, two attributes that can be antagonistic.

The difference between the utility function of the agent and of the principal can theoretically be detrimental to one party over the other. This would be the case, for example, in a situation where the principal asks for an HTA to legitimize the use of a technology, thus inciting through different communication signals the principal to be aware of this preference, but in trying to satisfy this principal’s preference, the agent may contribute to the reduction of its scientific reputation, an important attribute of its utility function. An LHTAU with a utility function that includes only one attribute—say scientific reputation (thus excluding the reputation of relevance)—could produce HTAs that would be detrimental to a decision maker who wishes to use the HTA recommendations to legitimize a technology, as in this case, the agent would not be willing to make a compromise that satisfies the strategic utility of the principal. In sum, the demand for HTAs within the health-care facility is possible in a context where the LHTAU has a dual-attribute utility function that makes it act to maintain both its scientific and relevance reputation.

### Empirical and theoretical regularities

In order to have a scientific explanation, these initial conditions should be combined with empirical or theoretical regularities.[7] First, there can be no communication or transmission of information between an agent and a principal when their preferences toward a state of the world differ too much.[9] This regularity has been tested multiple times in laboratory experiments.[4,10–13] Second, a principal will not delegate work to an agent with preferences that are too different from his own.[14] Third, overtime, workers in hierarchical organizations such as health care facilities tend to develop pride towards their practices[15] and to resist changes promoted by outsiders.[16] Finally, as already mentioned, health technology assessments have been shown to be used symbolically, that is to legitimize or delegitimize the use of health technologies.[17–21] /

## Materials and methods

### Data

We created a database of health technologies using the following inclusion criteria:

- assessed by the main LHTAU of the following university-affiliated health care centers: McGill, CHU de Montreal (CHUM), CHU de Québec and CHU de Sherbrooke;
- assessed in a formal, full-scale, health technology assessment report (excluding mini-HTA reports and knowledge syntheses) published on the LHTAU's web site from the date of creation of the evaluation unit until May 2016;
- assessed following an explicit demand from one or more individuals working inside the health care center as indicated in the HTA report (excluding technologies assessed following a demand from the outside);
- assessed in an HTA report that includes explicit recommendations regarding the relevance of using the technology;
- assessing the technology was an explicit objective indicated in the HTA report.

According to a recent mapping of resources in the Province of Quebec[22], 19 health care institutions have a health technology evaluation unit. For reasons of budgetary nature, it was not possible to study the reports of all 19 LHTAUs. We then chose to analyze the reports of the main LHTAU of each territory attached to one of the four faculties of medicine in Quebec, that is, the LHTAU of the Quebec City university health center (attached to Laval University), the Montreal university health center (attached to the University of Montreal), the McGill University Health Center, and the LHTAU of the Sherbrooke’s university health center.

The independence of the observations (i.e. the technologies) was ensured by excluding three technologies that have been assessed independently by two LHTAUs. When a given technology was assessed in two or more reports produced by the same LHTAU, we selected the most recent and up-to-date assessment.

### Data extraction and coding

The following information was extracted from the HTA reports by two independent coders (PL and AR) (verified by one auditor (MO)):

- name of the technology;
- type of technology (preventive, diagnosis, therapeutic or organizational);}
- name of the internal demander(s);
- name of the LHTAU;
- technology assessed in a report that also includes an assessment of one or more other technologies (yes or no);
- use status (technology already in use in the facility (yes or no));
- global efficacy judgment (authors believe that based on their review of the research evidence, the technology is effective, not effective or not enough supporting evidence—the last two categories were merged);
- direction of the recommendation regarding the relevance of using the technology (unfavorable or favorable).

The percentage of agreement between the two independent coders was 94.4 percent for the main explanatory variable (i.e. use status at the time of the evaluation) and 97.2 percent for the outcome variable (i.e. direction of the recommendation). Coding examples for the outcome variable are presented in Table 1.

**Table 1:** Coding examples for the outcome variable.

	Unfavorable recommendation	Favorable recommendation
In use within the facility at the time of the evaluation	Stop using TMIS or replace it with another or maintain use only as part of a research project. **Example:** "Treatment…remains an experimental procedure and needs further study… [The unit] offers an attractive recruitment potential for conducting these studies and an effort to obtain research funding for development is an avenue to explore."	Maintain routine use or maintain use in particular situations or extend use to other situations. **Example: "In conclusion**, the evaluation of the [unit] advocates maintaining diagnostic strategies in the detection of bone metastases in cancers…"
Not in use within the facility at the time of the evaluation	Do not integrate the technology or integrate it into a research project only. **Example:** "Routine use [of technology] for surgery […] should be re-examined as soon as the above data are available".	Integrating technology into routine practices or integrating technology into specific situations. **Example:** "As a result of the comments, observations and results of this study, we recommend that [the technology] become one of the working tools for the Service…"

As for the global efficacy judgment variable, we coded the sentences in the reports where the assessors summarize their interpretation of the research evidence that they found in their systematic literature review regarding the extent to which the technology succeeds in achieving its aim. Although all LHTAUs seem to use the same broad analytical approaches to determine efficacy (e.g. systematic review of RCTs), we noticed that the parameters used to form their final judgment of the available evidence are not standardized, meaning that they vary from one HTA report to the others. For example, the paucity of evidence on how to use a given technology (e.g. data on dosage) has been used to support the claim that there is not enough supporting evidence in favor of a technology, while in another report, the authors explicitly stressed the same gap in the evidence, but did not refrain from concluding that enough evidence exists in support of the technology.

Each technology in the database was classified in one of the following types: preventive, diagnosis, therapeutic or organizational. A preventive technology is a technology that aims at reducing the likelihood of adverse health outcomes (e.g. Safety devices to reduce needlestick injuries). Diagnosis technologies are those designed and used to identify the presence and nature of a health condition (e.g. Video Capsule Endoscopy Systems). Technologies aiming at treating a health condition were classified as therapeutic (e.g. Transcatheter aortic valve implantation). Finally, technologies that were classified as “organizational” are those that aim at improving one or more organizational or systemic aspects of health care (e.g. comprehensive policy for the antiviral treatment of hepatitis C patients, computerized prescription medical entry software, etc.).

### Data analysis

All data analyses were performed with Stata v12. The Stata file including the variables described below is available at: http://doi.org/10.3886/E100955V1. The research hypothesis was tested by estimating a logistic regression equation with the direction of the recommendation (i.e. unfavorable vs. favorable) as the outcome variable and the following variables as correlates:

- use status (0 = not in use in the facility at the time of the evaluation; 1 = in use in the facility at the time of the evaluation);
- global efficacy judgment (0 = authors believed that, based on their review of the research evidence, the technology is ineffective or there is not enough supporting evidence; 1 = the technology is considered effective);
- type of technology (one dummy per type of technology—therapeutic as the reference category);
- the LHTAU (one dummy per unit—McGill as the reference category);}
- technology assessed in a report that also includes an assessment of one or more other technologies (0 = no; 1 = yes).

We also estimated a logistic regression equation with the variable 'use status' as the outcome variable to examine the characteristics of the technologies that were in use in the facility at the time of the evaluation as compared to the technologies that were not in use. Rate ratios (RR) and their corresponding 95 percent confidence interval were calculated by using 'oddsrisk', a Stata module to convert Logistic Odds Ratios to Risk Ratios.[23] Each RR presented in the paper corresponds to the effect of one predictor of interest on the outcome variable. Each RR was calculated based on the prevalence rate of the predictor of interest (binary (1/0)).

## Results

### Sample characteristics

From the 108 health technologies included in our database, 63.9 percent (69) were recommended favorably by the LHTAU, while the use of 36.1 percent (39) were not recommended. Six of the 108 technologies were not explicitly assessed for their efficacy. From the 102 technologies that were assessed for efficacy, 61.8 percent (63) were judged as globally effective based on the review of existing research evidence, while 38.2 percent (39) were judged as either ineffective or as lacking proof of efficacy. A little more than half of the 108 health technologies were in use within the health care facility at the time of the evaluation (53.7 percent; n = 58), while 46.3 percent (50) were not in use. The majority of the technologies (59.3 percent; n = 64) were the only technology assessed in the HTA report.

The McGill HTA unit is the one that evaluated the largest number of technologies (44.4 percent; n = 48), followed by the CHU de Quebec (25 percent; n = 27), the CHU de Sherbrooke (17.6 percent; n = 19) and the CHUM (Montreal) (13 percent; n = 14). More than the half of the 108 technologies (53.7 percent; n = 58) were therapeutic, 18.5 percent (20) were preventive, 16.7 percent (18) were diagnosis technologies, and only 11.1 percent (12) were of the organizational type (e.g. new way of organizing services).

### Technologies' use status at the time of the evaluation

The technologies that were in use in the facility at the time of the evaluation were not more (or less) likely to be judged as effective by the assessors than the technologies that were not in use (RR = 1.41; 95 percent CI = 0.86–1.85). As compared with technologies that were not in use in the facility at the time of the evaluation, the technologies that were in use were more likely to be assessed in a report that also includes an assessment of one or more other technologies (RR = 1.68; 95 percent CI = 1.08–2.10). The technologies that were in use in the facility at the time of the evaluation were less likely to have been evaluated by the CHU de Sherbrooke's unit than by the McGill unit (RR = 0.30; 95 percent CI = 0.08–0.83) and of being of the organizational type rather than of the therapeutic one (RR = 0.17; 95 percent CI = 0.02–0.89)

### Are recommended technologies judged as more effective than unrecommended ones?

The answer to this question is clearly yes. More precisely, while controlling for the correlates described in the data analysis subsection and by using 'global efficacy judgment' as the primary predictor in the oddsrisk model, the technologies that were judged as effective by the assessors based on the review of existing evidence were much more likely to have been recommended favorably than those which were judged as ineffective or as lacking supporting evidence (RR = 3.28; 95 percent CI = 2.64–3.48).

### Do the assessors' recommendations vary according to the evaluation unit and the type of technology?

The logistic regression results show that, holding other things constant, one evaluation unit (i.e. the CHU de Sherbrooke) is more likely than the McGill one to recommend favorably the use of the technologies it assessed (RR = 1.72; 95 percent CI = 1.47–1.75). This evaluation unit has recommended favorably 16 of the 19 technologies it assessed (84.2 percent), while the positive recommendation rates for the other units were 63 percent (CHU de Quebec), 58.3 percent (McGill) and 57.1 percent (CHU de Montreal or CHUM).

As for the type of technology assessed, the regression results show that, holding other things constant, preventive technologies were less likely than the therapeutic ones to be recommended favorably (RR = 0.39; 95 percent CI = 0.09–0.98). A closer look at the raw data shows that the use of 72.4 percent of the 58 therapeutic technologies in the database was recommended favorably, as compared to 61.1 percent for the diagnosis technologies, 55 percent for the preventive technologies, and 41.7 percent for the technologies of the organizational type (although the chi-square test is not statistically significant: 5.15; p = 0.16, maybe due to the lack of statistical power).

### Should our research hypothesis be rejected?

The answer to this question is no, at least based on our data. Based on the principal-agent theory, we formulated the hypothesis that health technology assessment units located in health care facilities tend to be more optimistic toward technologies that are in use in their organization than toward technologies that are not. Controlling for the correlates described in the data analysis subsection and using the variable reflecting the use status as the predictor of interest in the oddsrisk model, technologies that were in use in the facility at the time of the evaluation are 62 percent more likely than technologies that were not in use to be recommended favorably by the assessors (RR = 1.62; 95 percent CI = 1.06–1.88).

The raw data show that the use of 75.9 percent (44) of the 58 technologies that were in use in the facility at the time of the evaluation was recommended favorably, while the use of 50 percent (25) of the 50 technologies that were not in use was recommended favorably (chi-square = 7.78; p = 0.005).

It should be recalled that the association between the use status of the technology within the facility at the time of the evaluation, and on the other hand the judgment in terms of efficacy is not statistically significant (chi-quare = 1.38, p = 0.24). Descriptive analyses also show that the use of 67.7 percent of the 28 technologies that the evaluators found to be effective, but which were not in use within the institution at the time of the evaluation, was favorably recommended, while 97.1 percent of the 35 technologies considered effective that were currently in use within the facility were favorably recommended, a difference of almost 30 percentage points.

Moreover, six of the 108 technologies have not been evaluated for their effectiveness. These six technologies were in use within the facility at the time of the evaluation and assessed in a report that also includes an assessment of one or more other technologies. The evaluators, without an explicit consideration of their efficacy, favorably recommended the use of five of them.

## Discussion

The results of a content analysis of 87 health technology assessment reports show a propensity of the LHTAUs to favorably recommend the use of a given technology when it is already in use in their facility. Interestingly, this optimism toward technologies that are already in use is observed in the four units considered (i.e. one unit was optimistic toward almost all technologies it assessed). It has also been shown that the greater propensity to recommend the use of a technology that is already in use in the facility at the time of the evaluation seems to be independent of evaluators' global judgment as to its efficacy.

Several mechanisms may explain the favorable bias of local HTA units with respect to technologies already in use in their facility. In this study, the principal-agent theory was used as a starting point for theorizing about the objectives and behavior of these units (agents). This theory allowed us to make some theoretical observations on the strategic-institutional context in which a local HTA unit operates. An HTA unit that is integrated within a health care facility owes its existence and survival to the level of demand for its assessment services. Considering the context of budgetary rationalization that prevails in the health sector and insofar as these units have an evaluation role and do not provide any essential management or clinical services, the survival and expansion of such units directly depend on their ability to stimulate demand for their services. As requests for health technology assessment are non-mandatory, the demand depends on the perceived usefulness of such services.

Health technologies used in healthcare facilities compete with other technologies available on the market. Adopting a new technology within an institution changes routines to which staff members may be attached. Like public servants, hospital staff are likely to identify themselves sentimentally with their lifestyles at work and may be led to develop a sense of pride in their practices and resist changes in routines, particularly the changes promoted by outside individuals or groups.[15] The propensity of the personnel of a hierarchical organization to protect themselves from external interference and to resist change has been emphasized for a long time (see, for example, [16]).

There are some limitations to our study that deserve to be recalled. First, the results obtained apply to the four main local HTA units in Quebec and cannot be generalized to all local HTA units in Quebec, in Canada or elsewhere in the world. However, the theoretical elements of the principal-agent theory with the empirical results allow us to believe, until the contrary is shown, that the optimism of local HTA units with regard to the technologies already in use within their institution could prevail beyond the population studied. In effect, regardless of the health system in which it operates, any HTA unit that carries the mission of assessing technologies on behalf of stakeholders from the same organization lacks the structural independence that would protect it from internal vested interests. This situation theoretically applies to local HTA units in other Canadian provinces and other countries. A second potential limitation of the study is the possibility that there were cases where the local HTA unit has chosen not to publish the report in order not to harm the strategic interests of the demander. We contacted the four units to know whether they publish all their reports on their website. Three of the four units replied to our information request. These three units declared that all of their reports were made available on their web site.

This study is only the first step in a larger undertaking. First, a next step could be to extend the analysis to other local HTA units to increase the number of observations and hence the statistical power. This would allow us to integrate additional variables into the analysis, such as the assessors' judgment regarding adverse effects and economic evaluation. Second, a qualitative case study of some evaluations with inconsistencies between the scientific evidence and the recommendations would also be carried out in order to analyze more closely the structure of the arguments put forward by the evaluation units to support their recommendations regarding the use of health technologies within their institution. The current trend towards the multiplication of formal criteria mobilized by evaluation units to formulate their conclusions and recommendations, that is, the addition of criteria beyond the traditional parameters of clinical efficacy and safety, increases the number of arguments that can be mobilized to recommend the use of a given technology. Analyzing more closely the discourse of the evaluation units, it would thus be possible to identify the main language games used in the HTA reports produced by local evaluation units. While coding the reports to create the variable capturing the assessors’ global judgment of the research evidence concerning the efficacy of the technologies, we noticed that this interpretation of the evidence base does not seem to be based on standardized parameters. For example, the same type of gap in the research evidence (e.g. absence of data on dosage) does not seem to have the same effect on assessors’ judgment across different health technology assessments. This flexibility in the synthesis of the evidence provides the assessors with the opportunity to inflate or disinflate the efficacy of a given technology, a phenomenon that was observed in scientific publications.[24] Furthermore, the fact that the use status of a given technology at the time of its evaluation is a significant predictor of the direction of the recommendation, but not of the assessors’ global judgment regarding its efficacy, suggests the use of a non-standardized and flexible approach to recommendation formulation and justification, a phenomenon that we will investigate by qualitative content analysis of the recommendation section of HTA reports.

In future research, one could extend the analysis to HTAs produced by provincial and national HTA units to see if they tend, like the local HTA units considered in this study, to be more optimistic toward technologies that are already in use by the organizations that requested the assessments. What is also needed is theoretical and empirical research on institutional arrangements regarding the production of HTAs. For example, should health technology assessments be produced in-house? Which institutional arrangements would be better suited to protect the independence of HTA units and reduce the possibility for both HTA units and decision makers to act strategically? It could be the case that institutional arrangements regarding HTAs influence the content of the utility function of both the HTA units and the decision makers. Should we design institutional arrangements so that HTA units would act as if they have a single-attribute utility function aimed at securing its scientific reputation without having to bother about its survival and growth? These questions were beyond the scope of this study and need further investigation.

Finally, another research avenue to be explored is that the age of a technology, that is, the number of years since its introduction on the market, could increase the likelihood that it will be favorably recommended, following a lock-in effect similar to that modeled by Arthur.[25] Adding the age of technologies to an expanded database will allow testing this hypothesis. In the event that we find that the age of health technologies is associated with the nature of local HTA units' recommendations, then we would be led to re-examine the subject matter from a historical and evolutionary perspective, not exclusively centered on the strategic elements proper to the principal-agent theory.
